# Supplementary material for: Examining the Double Burden of Underweight, Overweight/Obesity and Iron Deficiency among Young Children in a Canadian Primary Care Setting
Source: Nutrients. 2023 Aug 18;15(16):3635. doi: 10.3390/nu15163635 (PMC10458882; doi:10.3390/nu15163635)
Supplement: Supplementary file 1 [file nutrients-15-03635-s001.zip › nutrients-2561784-supplementary.pdf]

Table S1. Characteristics of children with data on zBMI compared with children who had missing data on.zBMI (n=208).

Characteristics	Response Sample		Children with Missing zBMI Data	
	n	Mean (SD) or N (%)	n	Mean (SD) or N (%)[1]
**Patient-level characteristics**				
Child age, months	1953	18.3 (5.0)	208	17.9 (5.3)
Child sex, Female	1953	945 (48.4)	208	91 (43.8)
zBMI	1953	0.13 (1.1)	0	–
Weight category	1953		0	
Underweight (zBMI < -2)		51 (2.6)		–
Normal weight (-2 ≤ zBMI ≤1)		1495 (76.6)		–
At-risk-of-overweight (1 < zBMI ≤ 2)		312 (16.0)		–
Overweight/obese (zBMI > 2)		95 (4.9)		–
Birthweight, kg	1835	3.3 (0.6)	168	3.3 (0.6)
Maternal ethnicity[2]	1725		144	
European		1112 (64.5)		84 (58.3)
Non-European		613 (35.5)		60 (41.7)
Maternal education	1763		130	
High school or less		141 (8.0)		13 (10.0)
College/University		1622 (92.0)		117 (90.0)
Family income (CAN $)	1604		98	
Less than $40,000		161 (10.0)		16 (16.3)
$40,000 - $79,999		227 (14.2)		9 (9.2)
$80,000 - $149,999		546 (34.0)		25 (25.5)
$150,000 +		670 (41.8)		48 (49.0)
**Infant feeding practices**				
Breastfeeding duration ≥12 months	1640	869 (53.0)	128	59 (46.1)
Bottle use >15 months	1612	480 (29.8)	114	32 (28.1)
Daily cow's milk intake >2 cups (500mL)	1620	422 (26.1)	122	28 (23.0)
**Laboratory characteristics**				
Serum ferritin (μg/L)	1953	27.4 (18.7)	208	28.8 (19.7)
Iron deficiency	1953	269 (13.8)	208	25 (12.0)

[1]Data are presented as mean (SD) or N (%). [2] Maternal ethnicity: European includes Western European, Eastern European, and Australian or New Zealander; Non-European includes East Asian, Southeast Asian, South Asian, West Asian, African, Caribbean, Latin American, North American Indigenous, and Mixed (2 or more ethnic groups). .
